# Three-dimensional single-cell imaging with X-ray waveguides in the holographic regime

**DOI:** 10.1107/S2053273317007902

**Published:** 2017-06-29

**Authors:** Martin Krenkel, Mareike Toepperwien, Frauke Alves, Tim Salditt

**Affiliations:** a Institut für Röntgenphysik, Georg-August-University Göttingen, Germany; b Max-Planck-Institute for Experimental Medicine and University Medical Center Göttingen, Germany

**Keywords:** X-ray holography, phase retrieval, X-ray tomography, X-ray waveguides, coherent imaging

## Abstract

X-ray tomography at the level of single biological cells is possible in a low-dose regime, based on full-field holographic recordings, with phase contrast originating from free-space wave propagation. Building upon recent progress in cellular imaging based on the illumination by quasi-point sources provided by X-ray waveguides, here this approach is extended in several ways. First, the phase-retrieval algorithms are extended by an optimized deterministic inversion, based on a multi-distance recording. Second, different advanced forms of iterative phase retrieval are used, operational for single-distance and multi-distance recordings. Results are compared for several different preparations of macrophage cells, for different staining and labelling. As a result, it is shown that phase retrieval is no longer a bottleneck for holographic imaging of cells, and how advanced schemes can be implemented to cope also with high noise and inconsistencies in the data.

## Abstract 2

Phase-contrast X-ray imaging of biological cells in two and three dimensions can be carried out with a low dose, based on free propagation and a setting of optimized wavefronts in cone-beam geometry. In order to reach the required contrast level, images have to be recorded in the holographic regime. The main result of this work is holographic recordings of a quality that is fully amenable to quantitative phase retrieval, beyond previous approximations. Different approaches to sample preparations, data recording and phase retrieval are compared.

## 1. Introduction   

Imaging and tomography of biological cells with hard X-rays are associated with considerable challenges. For these weakly diffracting objects, it is much more difficult to reach high resolution and sufficient contrast than for most other samples. At the same time, hard X-rays can in principle probe the cell’s native electron-density distribution at subcellular resolution with quantitative contrast (Wilke *et al.*, 2015 ▸), provided that the signal-to-noise ratio is sufficient. Furthermore, penetration power and depth of focus enable studies of cells embedded in complex environments as well as cells enclosed deeply within tissue (Krenkel *et al.*, 2015 ▸). In this way, hard X-ray imaging can complement established imaging techniques such as fluorescence light microscopy, electron microscopy and soft X-ray microscopy (Larabell & Nugent, 2010 ▸).

Indeed, recent progress in lensless coherent X-ray diffractive imaging (CDI) has enabled several impressive two-dimensional and three-dimensional studies of subcellular architectures (Shapiro *et al.*, 2005 ▸; Song *et al.*, 2008 ▸; Nishino *et al.*, 2009 ▸; Lima *et al.*, 2009 ▸; Huang *et al.*, 2009 ▸; Giewekemeyer *et al.*, 2010 ▸; Wilke *et al.*, 2012 ▸). This CDI approach based on far-field coherent diffraction will certainly undergo further progress. However, these studies are usually operated in a regime of high radiation dose, typically in the range of 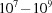
 Gy, creating a need for cryogenic conditions to ensure structure preservation. This dose problem has been a concern in far-field diffractive imaging, which has persisted from the original approach based on plane-wave illumination and oversampling (Miao *et al.*, 1999 ▸), to the more recent ptychographic phase retrieval based on lateral scanning with an overlap between successive exposures (Thibault *et al.*, 2008 ▸). In this work we explore phase-contrast imaging of biological cells in the low-dose regime without cryogenic preservation, based on full-field propagation imaging in cone-beam geometry. Specifically, we want to investigate whether subcellular details are still observable at doses in the range 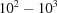
 Gy (per projection), at least for stained and contrasted samples, and which phase-retrieval algorithms are best suited for this purpose.

As is well known, propagation-based phase contrast enables X-ray imaging of weakly absorbing specimens (Paganin, 2006 ▸). Since it is a full-field approach, it is particularly well suited for tomography and for dynamic studies. It is also dose efficient, as no optical element is placed between the sample and the detector. And finally, using cone-beam geometry, the field of view and magnification can be conveniently adapted by the change of the source-to-sample distance. However, in most cases phase-contrast experiments are carried out in the direct-contrast regime, where the full potential of the intrinsic phase sensitivity is not exploited (Burvall *et al.*, 2011 ▸). An increase of the (effective) propagation distance brings the measured intensity distributions to the more sensitive holographic regime (Bartels *et al.*, 2015 ▸). In this regime phase retrieval is a necessary step to obtain information about the object’s exit wave and hence the structure of the object. Furthermore, the illumination needs to be well known in this regime in order to ensure meaningful phase reconstructions. If significant artifacts disturb the wavefront (probe), near-field ptychography can be used to reconstruct object and probe (Robisch *et al.*, 2015 ▸; Stockmar *et al.*, 2015 ▸), at the expense, however, of additional exposures and therefore also dose. This may limit the applicability to radiation-sensitive samples like biological tissue.

Here we use X-ray waveguides to provide a highly coherent, well controlled and quasi-point-like illumination (Osterhoff & Salditt, 2011 ▸; Krenkel *et al.*, 2015 ▸; Bartels *et al.*, 2015 ▸) for tomographic studies at the level of single cells. Compared with earlier work on cellular tomography of bacterial cells (Bartels *et al.*, 2012 ▸), we here demonstrate a significantly enhanced three-dimensional image quality allowing for the identification of much more interior structural details, while simultaneously increasing the field of view. This is demonstrated for macrophages as examples of larger eukaryotic cells. The demonstrated progress in image quality has been obtained *via* improvements on different levels, starting from the waveguide optics, the alignment and imaging processing procedure, the recording and detection scheme, and finally the reconstruction. To this end, we first provide a thorough study of phase-retrieval techniques for the holographic regime, including a recent approach based on an iterative regularized Gauss–Newton method (Maretzke *et al.*, 2016 ▸), and also a novel variant of the so-called holo-TIE reconstruction (Krenkel *et al.*, 2013 ▸). Importantly, this holo-TIE approach put forward here for tomography of single cells does not rely on assumptions on material composition nor on linearization of the specimen’s optical constants, as is typically the case in conventional non-iterative reconstruction schemes.

## 2. Data recording and processing   

Before turning to the experimental details we will hence first address the phase-retrieval approaches used in this paper. Note that these approaches are applicable to any propagation-based phase-contrast experiment, independent of the specific recording geometry, such as plane-wave or cone-beam illumination. For example, they could equally well be applied to propagation imaging with visible light, neutrons or electrons.

### 2.1. Phase-retrieval algorithms   

We start with the Helmholtz equation in scalar and paraxial approximation (Goodman, 2005 ▸) to describe the spatial evolution of the electromagnetic X-ray field. Given a complex valued solution 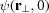 in a plane at 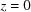, the wavefield after a propagation distance *z* in vacuum (and in excellent approximation also in air) can be described by the so-called Fresnel propagator 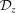 (Paganin, 2006 ▸), 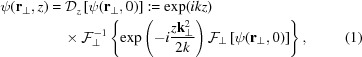
where 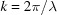 denotes the wavenumber for wavelength λ and 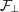 the lateral Fourier transform with respect to 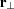, with 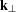 as the reciprocal coordinate vector. This equation can be used to numerically simulate free-space propagation experiments and, further, to provide a very basic reconstruction by numerical back-propagation of the measured intensity distributions 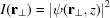. The back-propagated field 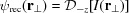 will contain information about the exit plane of the original object, superimposed with the so-called twin image (Gabor, 1948 ▸), which is present due to the lack of information about the detector phase distribution. This reconstruction is known as *holographic reconstruction* and typically used for reference beam holography, where the twin image is spatially separated in the reconstruction due to a slight angle between object and reference beam (Gauthier *et al.*, 2010 ▸).

Another way to describe the spatial evolution of intensity distributions is based on the transport of intensity equation (TIE) (Teague, 1983 ▸): 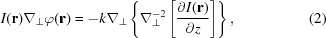
which is derived from the paraxial Helmholtz equation. For short propagation distances and the assumption of a phase-attenuation duality, as applicable to single-material objects, a direct reconstruction formula for the phase of the object exit plane can be derived (Paganin *et al.*, 2002 ▸), 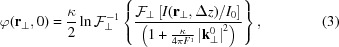
where the geometric parameters have been condensed to the Fresnel number for a single pixel 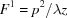 with the pixel size *p*, leading to unitless reciprocal coordinates 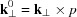. κ describes the ratio between the decrement and imaginary part of an (effective) refractive index 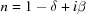. In practice, the deterministic ratio 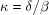 is often replaced by a parameter that is chosen based on visual inspection of the reconstructed image. There are similar TIE-based approaches, which lead to slight differences in the implementation (Burvall *et al.*, 2011 ▸). However, all of them have in common that they rely on short propagation distances, *i.e.* large Fresnel numbers. The algorithm defined by equation (3) will be referred to as *Paganin phase retrieval*.

For smaller Fresnel numbers, *e.g*. in the holographic regime which is favoured in view of its stronger phase effects (contrast) in the measured image, another phase-retrieval approach is commonly used, which also relies on Fourier filtering (Zabler *et al.*, 2005 ▸; Cloetens *et al.*, 2006 ▸; Langer *et al.*, 2008 ▸). It is based on the contrast transfer function (CTF) (Guigay, 1977 ▸), which provides a linearization of the image formation also for smaller Fresnel numbers as long as the objects are ‘weak enough’, *i.e.* as long as the object exhibits a slowly varying phase and weak absorption. In this case, a deterministic phase-retrieval approach based on the known functional form of the CTF can be derived (Cloetens, 1999 ▸). For the aforementioned phase-attenuation duality, least-square minimization of the CTF leads to (Turner *et al.*, 2004 ▸; Gureyev *et al.*, 2004 ▸) 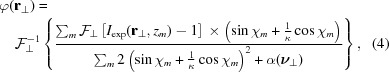
with an additional frequency-dependent regularization 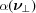 of high spatial frequencies and 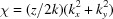 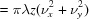, where 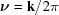 are the spatial frequencies, occurring when the Fourier transform without prefactors is used. By introducing multiple measurement planes at distances 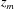, the influence of zeros in the CTF can be reduced. Here we refer to algorithms based on equation (4) as *CTF-based phase retrieval*. A very similar approach is the quasi-particle approach, which was introduced as an extension beyond the linear approximations in Moosmann *et al.* (2010 ▸). Instead of adding an additive regularization, spatial frequencies close to the singularities in the CTF are suppressed by masking them out. In practice, almost no difference between both methods can be observed if the regularizing parameter α is chosen correctly.

To account for the full non-linear image formation, necessary *i.e.* for samples that strongly violate the assumptions used for the derivation of the CTF, iterative algorithms are the method of choice. These are based on sequential numerical back- and forward-propagations of the wavefunction, where the measured intensity distribution in the detector plane is enforced and additional *a priori* information in the object plane can be easily incorporated (Fienup, 1982 ▸; Latychevskaia & Fink, 2007 ▸). Typical object-plane constraints are the assumption of a pure-phase object, positivity of electron density or absorption and the presence of a compact support of the object, *i.e.* the object is limited to a finite region in real space. A *modified hybrid input–output algorithm* (mHIO), where these three constraints are applied, has turned out to provide excellent results on isolated weak objects and it is able to reconstruct even those spatial frequencies that are not transmitted due to the zeros of the CTF (Giewekemeyer *et al.*, 2011 ▸). Here, we generalize the method to include multiple measurement planes, such that the iterations are carried out sequentially for each plane. Further, soft projections are used as described in Giewekemeyer *et al.* (2011 ▸) to compensate non-perfect experimental conditions. As a rather new approach for iterative phase retrieval, we also use the iteratively regularized Gauss–Newton (IRGN) method in this work. The IRGN approach differs from the widespread alternating-projection-type algorithms in that it exploits differentiability and simultaneously processes constraints and observed data, resulting in improved convergence (Maretzke *et al.*, 2016 ▸). Mathematically, IRGN is a Tikhonov regularized version of a Newton-type iterative solution, 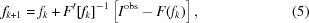
where *F* denotes the (non-linear) forward operator and 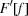 its Fréchet derivative (Hohage, 1997 ▸). Note that the linearization is local with respect to the current iterate 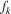 and thereby better justified than static linearization as in the CTF approach. In contrast to Maretzke *et al.* (2016 ▸), where IRGN was used for single-distance recordings, we have implemented it here also for multiple-distance data sets, in order to provide a valid comparison with CTF phase retrieval and holo-TIE (Krenkel *et al.*, 2013 ▸).

Similar to CTF, holo-TIE is a deterministic inversion based on a multiple-distance data set, treated by Fourier methods, but without linearization of the object’s optical constants (Krenkel *et al.*, 2013 ▸). Thus, no restrictive assumptions on the object have to be made. In its standard version, it uses two slightly different object-to-detector distances (effective defocus distances), with intensity images 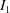 and 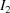, and difference image 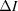 to calculate the phase distribution 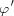 in the detection plane, as originally proposed by Paganin & Nugent (1998 ▸), 
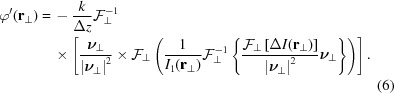
With the complete wavefunction in the detector plane at hand, the field can be back-propagated to the object plane. This so-called *holo-TIE* reconstruction can be calculated in natural units of pixel size and Fresnel number, as for the algorithms presented above. To this end the prefactor 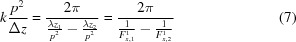
is rewritten and normalized spatial frequencies 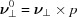 are introduced. However, photon noise poses an experimental limitation to the determination of the difference image 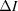, which should approximate the derivative. To overcome this limitation, we here put forward a further refinement of this approach, using additional knowledge from multiple measurements or reconstructions. The starting point of this modification is based on the observation that high spatial frequencies of the recorded image evolve quickly along the propagation direction *z*, suggesting the use of a small 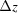 for the measurement. The low spatial frequencies are more stationary with *z*, requiring a larger 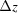 in order to obtain a robust measurement of the differential quotient as an estimator for the intensity derivative. Hence, the idea is to combine both cases in multiple measurements and to take the respective frequency region in Fourier space that is well transferred.

**Figure 1:**
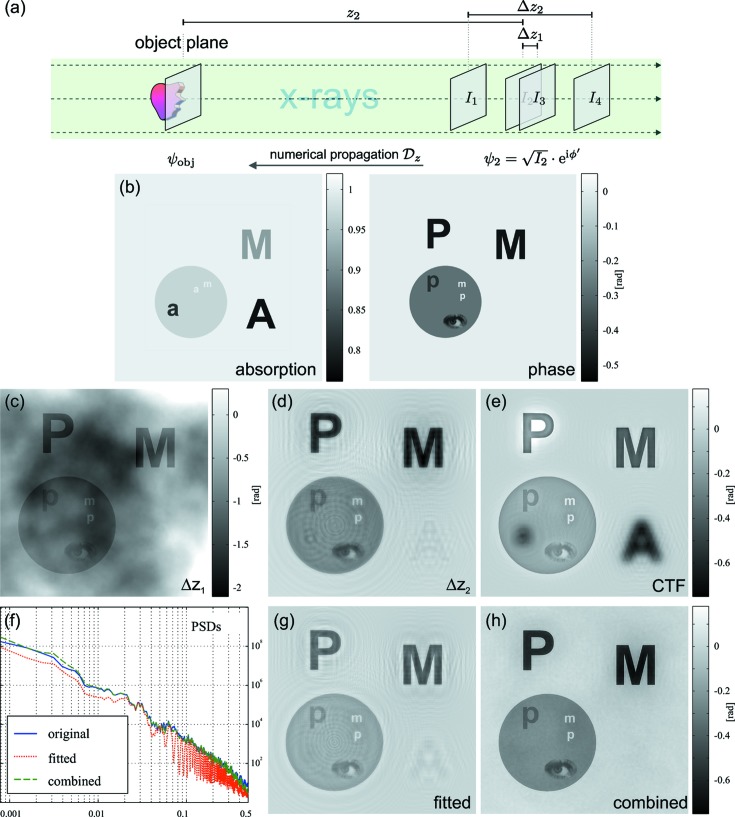
Improved holo-TIE scheme. (*a*) Sketch of the algorithmic concept. Multiple images with different Fresnel numbers are recorded, *e.g.* by changing propagation distances. Distances are chosen such that two images have a small distance 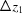 and two other images have a larger distance 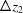. (*b*) The phantom consists of different image components: (A) absorption contrast with 0.76 transmission, (P) phase contrast with 0.55 rad phase shift and (M) mixed contrast of both. (*c*) Reconstructed phase of the simulated test object (see main text) in the object plane using two images separated by 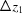. (*d*) Reconstructed phase in the object plane using two images separated by the larger 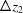. (*e*) Phase distribution retrieved using the CTF with homogeneity assumption. Pronounced artifacts near the image components representing pure phase contrast (P) and pure absorption (A), respectively, are observed. (*f*) Power spectral densities of the original object compared with the multi-distance holo-TIE reconstructions. (*g*) Multi-distance holo-TIE reconstruction as described in Waller *et al.* (2010 ▸). (*h*) Combined multi-distance holo-TIE approach where the detector-plane phase is obtained by a weighted combination of the detector phases from (*c*) and (*d*).

Fig. 1 ▸ shows illustrative reconstructions of a simulated multi-material object (phantom) together with a sketch of how images are arranged in the optimized holo-TIE scheme of this work. The phantom comprises purely phase shifting structures (P’s and the eye), purely absorbing structures (A’s) and mixed objects (M’s and the circle). Thus, conventional reconstructions, *e.g.* based on the CTF with homogeneity assumption, cannot be expected to perform well. A total of four noisy intensity distributions have been simulated such that the images 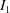 and 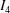, separated by 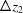, have a large Fresnel number difference, while the images 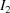 and 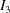, separated by 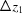, differ only slightly in their Fresnel number. In order to approximate the differential quotient sufficiently well, the Fresnel numbers of the two recorded images have to be approximately equal, *i.e.* ideally 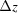 has to be vanishingly small. However, in the presence of noise, 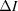 will then suffer from low signal-to-noise, resulting in artifacts [see the cloudy noise in Fig. 1 ▸(*c*)]. Increasing the difference 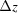 reduces the susceptibility to noise, but now the differential quotient is poorly approximated by the difference quotient, resulting in high-frequency artifacts (Fig. 1 ▸ *d*). For comparison, a CTF reconstruction with homogeneity assumption is shown (Fig. 1 ▸ *e*), which also fails to deliver satisfactory results, as expected. Further, Fig. 1 ▸(*g*) shows a reconstruction based on an improved approximation of the differential quotient by polynomial fitting, as described in Waller *et al.* (2010 ▸). This method reduces the influence of noise and manages to slightly reduce the high-frequency artifacts, but as only four images are available here, the resulting object-plane phase distribution still shows deviations from the phantom (ground truth). Finally, we have implemented a novel scheme, where the distances 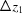 and 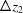 are optimized, such that one pair provides a slight Fresnel number difference and the other a larger one. Two independent phase distributions in the detector plane 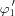 and 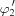 are reconstructed according to equation (6) for the distances 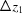 and 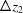, respectively. A combination 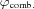 of both phase maps is created by taking high spatial frequencies solely from 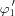 and low spatial frequencies from 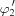. The combination is implemented by 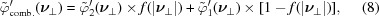
where 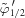 denotes the Fourier transform of the respective phase distribution in the detector plane. By a suitable choice of *f*, a smooth transition can be realized, *e.g.* by the point symmetric two-dimensional error function 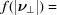 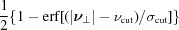. The transition parameters are chosen to 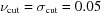
 periods per pixel, based on visual inspection of the resulting phase map. Fig. 1 ▸(*h*) shows that the resulting phase distribution has almost no deviations from the ground truth, which is further corroborated by the averaged power spectral densities shown in Fig. 1 ▸(*f*).

The last phase-retrieval approach to be applied in this paper is a combined three-dimensional reconstruction. It is motivated by the fact that neighbouring projections are correlated in low spatial frequencies, which can be used to stabilize iterative phase retrieval (Ruhlandt *et al.*, 2014 ▸). Here, it is implemented as an extension to the classical multiplicative algebraic reconstruction technique (mART) (Gordon *et al.*, 1970 ▸). Briefly, update factors are calculated that compare the currently guessed object exit plane 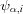 with a projection to the measured data 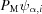, 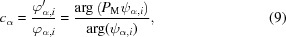
where 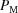 denotes the modulus constraint. These update factors are back-projected to a three-dimensional volume and multiplied with the current three-dimensional guess 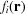, which is reprojected to yield a new guess for the exit plane: 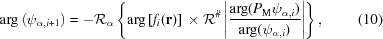
where 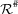 denotes the full back-projection operator and 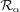 denotes the forward-projection operator for a single angle α. The same scheme can be applied to the modulus of the wavefunction if absorbing objects are to be reconstructed. For pure-phase objects, the modulus is set to unity.

### 2.2. Data recording and pre-processing procedures   

**Figure 2:**
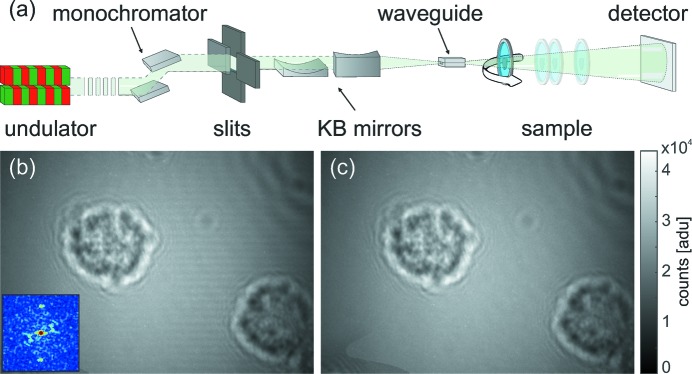
(*a*) The generic synchrotron setup used for waveguide-based X-ray imaging: monochromatic X-rays are pre-focussed to increase the efficiency of the waveguide coupling. Following the propagation in the waveguide, a well defined divergent X-ray beam emanates from the exit plane. Samples are placed in a defocus plane at some distance from the waveguide exit for the recording of holographic images. (*b*) Measured raw data showing a hologram of two single macrophages, superimposed with the structure of the camera flat field and periodic stripes, which are caused by the waveguide. The inset shows the central part of the power spectral density (PSD) (log scale). (*c*) Raw hologram after stripe removal as described in the main text.

All of the experimental data shown in this paper have been recorded at the GINIX endstation of the coherence beamline P10 at PETRA III in Hamburg, Germany. The generic setup is sketched in Fig. 2 ▸(*a*) and described in detail in Salditt *et al.* (2015 ▸). An X-ray waveguide has been used as the beam-defining optics. This has the advantage that the illumination is nearly free of high-frequency artifacts, which is a key point for holographic X-ray imaging (Hagemann *et al.*, 2014 ▸). Further, the source size is reduced to below 50 nm (Bartels *et al.*, 2015 ▸) and the degree of coherence is increased (Osterhoff & Salditt, 2011 ▸). However, waveguides based on sputtered multilayer structures, as described in Krüger *et al.* (2012 ▸), sometimes exhibit residual periodic *stripe artifacts* in the illumination, as can be seen in Fig. 2 ▸(*b*). These high-frequency features in the illumination do not fully cancel during empty-beam division even if they are temporally stable. Contrarily, the artifact in the upper-right corner perfectly cancels in empty-beam division, as it results from a faulty scintillator, and hence is not subject to any propagation.

**Figure 3:**
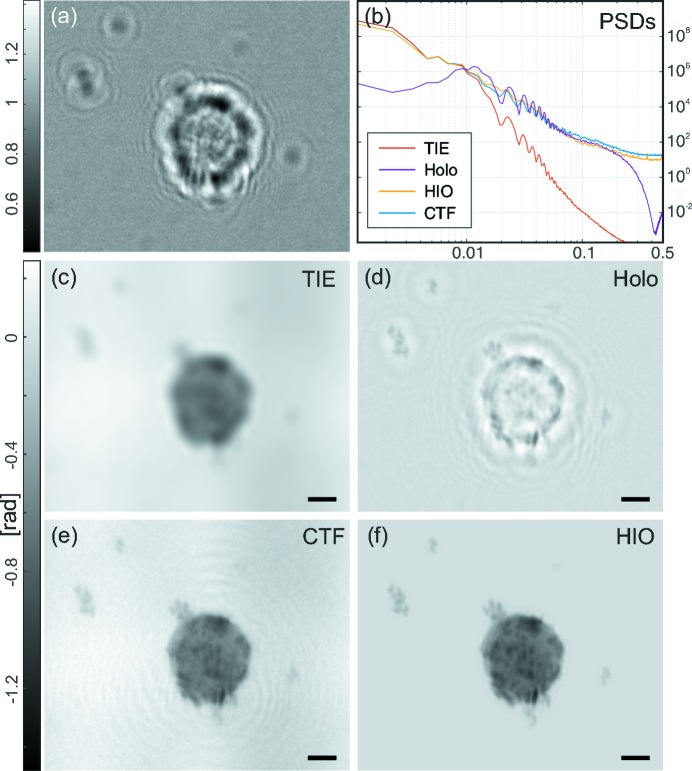
Single-distance phase-retrieval approaches to cellular imaging. (*a*) Processed hologram of a single macrophage, *i.e.* showing the raw hologram after stripe removal, empty-beam correction and background equalization. (*b*) PSDs of all following reconstructions. (*c*) ‘Paganin’ reconstruction based on the near-field TIE. (*d*) Holographic reconstruction based on back-propagation of the measured intensities. (*e*) CTF-based reconstruction with homogeneity assumption. (*f*) Iterative HIO reconstruction with automatically determined support and pure-phase constraints. All scale bars denote 5 µm.

**Figure 4:**
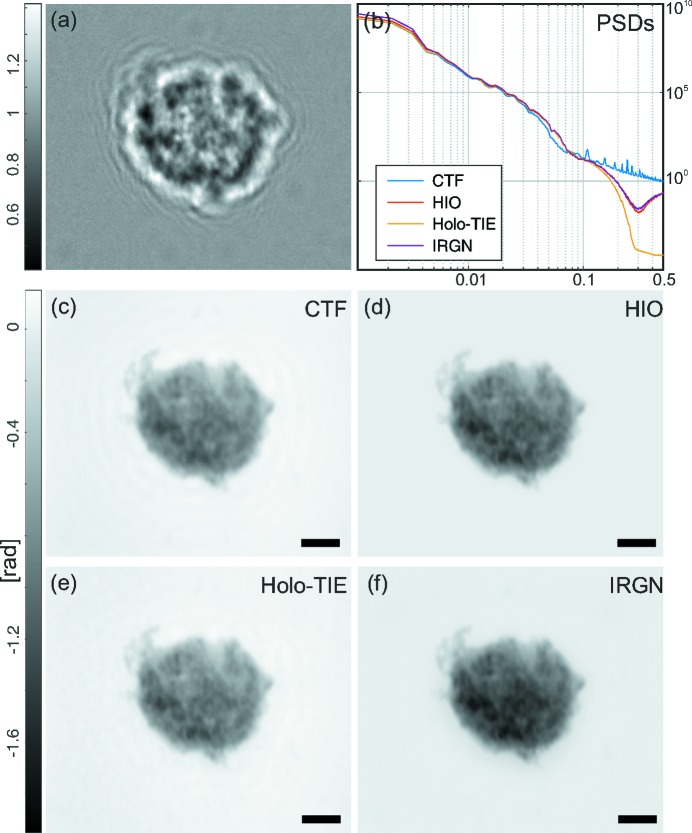
Multi-distance phase retrieval. (*a*) One representative example out of four processed holograms of a single cell in a defocus series. (*b*) PSDs of all following reconstructions. (*c*) CTF reconstruction using all four distances and a homogeneity assumption. (*d*) Iterative HIO reconstruction with support and pure-phase constraints. (*e*) Phase distribution in the object plane retrieved with the combined holo-TIE scheme using two intensity images, where the low-frequency image was created using the CTF guess. (*f*) IRGN reconstruction using all four measurement planes with a pure-phase assumption, but without any support constraint. From Krenkel (2015 ▸). All scale bars denote 5 µm.

To remove the stripe artifacts from the projections, a Fourier filter mask has been used. The inset in Fig. 2 ▸(*b*) shows the central part of the power spectrum of the raw hologram shown. Two distinct peaks can be identified corresponding to the periodic stripe artifacts. As the stripe direction does not change over time, they can be easily removed by an appropriate mask, replacing the masked (complex-valued) pixels with an average of pixels of the same spatial frequency. Fig. 2 ▸(*c*) shows the result of this stripe-removal approach. After the stripe removal, which is performed on the raw data, classical empty-beam correction is performed by subtracting a dark image with no radiation, followed by division by an empty-beam image, for which the sample is moved out of the beam. In some cases, a low-frequency variation in the images can be observed caused by slight changes in the illumination. These are suppressed by calculating correction profiles at the edges of the image like in Giewekemeyer *et al.* (2011 ▸), to obtain the finally corrected holograms. Two representative examples are shown in Figs. 3 ▸(*a*) and Fig. 4 ▸(*a*). More details regarding experiment and analysis of this work are given in the thesis of the first author (Krenkel, 2015 ▸).

### 2.3. Preparation of biological cells   

We have used stained macrophages from the mouse alveolar macrophage cell line MH-S as a test sample and to demonstrate the proof-of-concept reconstructions. Cells were cultivated as described in Krenkel *et al.* (2015 ▸). Macrophages were cultivated in RPMI medium (Gstraunthaler & Lindl, 2013 ▸), supplemented with 10% foetal calf serum (FCS), 0.05 m*M* 2-mercaptoethanol and 2.06 m*M* glutamine in a humidified atmosphere with 5% CO_2_ concentration at 310 K. Depending on the experiment, the cells were either cultivated in a Petri dish, or on thin plastic membranes, or in cell flow chambers (ibidi GmbH, Germany), or were embedded in agarose. In a first approach, to study the influence of different phase-retrieval algorithms, dried cells were prepared on 1 µm-thin plastic slides (MMI MembraneSlides, MMI, Germany). To this end, cells were chemically fixed [0.2% glutaraldehyde and 2% paraformaldehyde (PFA)] and dehydrated in air at room temperature. Second, we have used a resin-embedding protocol known from electron microscopy, which is expected to better preserve the structure. Samples were embedded in 2 mm-thick sticks of resin. Third, to study the cells in a hydrated environment, cells were placed in agarose gel. Finally, as the most structure-preserving preparation, we used a liquid chamber (μ-Slide, ibidi GmbH, Germany), in which the cells adhere to the surface of the chamber.

For embedding in agarose, cells were centrifuged and resuspended in 1% low-melting agarose (NuSieve GTG agarose, FMC Bioproducts) in 4% PFA in phosphate-buffered saline (PBS), kept in liquid state at 315 K. The cellular suspension was transferred to glass capillaries (Hilgenberg, Germany) by capillary forces (suction), and by cooling to room temperature below the gel transition the position of cells was fixed. For embedding in resin, the cells were dehydrated by an ascending series of ethanol and washed three times with 100% ethanol. Then, liquid Epon (Serva, Germany) was used for ethanol replacement in an ascending series, with 20 min incubation after each step, ending with 100% Epon. After the full substitution, the sample was baked for 24 h at 333 K, and samples were cut into pieces and polished.

For staining with BaSO_4_, 6 million cells were incubated for 4 h in 3 ml medium with 1–10 µl of a BaSO_4_ suspension (50 mg ml^−1^), commercially available as a radiography contrast agent (Microopaque CT, Belgium). For staining with osmium tetroxide (OsO_4_), a 2% OsO_4_ solution in PBS was applied for 4 h before the ascending dehydration series and the final sample was fixed in a resin block. For labelling with antibodies conjugated to nanogold, cells were cultivated in a cell flow chamber (ibidi GmbH, Germany) or on a membrane slide, fixed with formaldehyde and washed with PBS. Subsequently, the cells were permeabilized using 0.2% Triton X-100 and 1% normal goat serum (NS) in PBS for 5 min on ice, followed by washing with 1% NS in PBS three times. Cells were incubated with the primary antibody (rabbit anti-CD68) for 1 h at room temperature, followed by another washing with 1% NS and 5% bovine serum albumin (BSA) in PBS. 10 µg ml^−1^ of the secondary antibodies (Alexa Fluor 594 fluoronanogold) were diluted in PBS with 1% BSA incubated for 1 h in the dark, followed by washing with pure PBS. Finally, for the gold enhancement, the cells were washed and treated with a gold-enhancement kit (Gold Enhance EM Plus, Biotrends).

More details regarding the entire preparation and mounting of the cells can be found in Krenkel (2015 ▸).

## 3. Experimental results   

### 3.1. Two-dimensional phase retrieval   

Fig. 3 ▸ shows the performance of different reconstruction methods for single-distance phase retrieval of single macrophage cells. The empty-beam corrected hologram shown in Fig. 3 ▸(*a*) is used for four different phase-retrieval approaches. In Fig. 3 ▸(*b*) a comparison of the four reconstructions in Figs. 3 ▸(*c*)–3 ▸(*f*) is shown in Fourier space, where an angular average was used to reduce the data of the power spectrum to a one-dimensional curve. As the Fresnel number is too small to warrant the direct-contrast regime, the TIE reconstruction shown in Fig. 3 ▸(*c*) yields a blurred reconstruction. The reconstruction approximates only the low spatial frequencies of the cell. The holographic reconstruction of the macrophage (Fig. 3 ▸ *d*) is superimposed by the typical twin image artifact, resulting in a loss of quantitative contrast values. The CTF-based phase retrieval in Fig. 3 ▸(*e*) yields a reasonable reconstruction. However, some residual twin image artifacts can still be recognized. This is due to the fact that some spatial frequencies are not transmitted, as predicted by the CTF. The best reconstruction is obtained with the iterative mHIO approach, shown in Fig. 3 ▸(*f*). Due to the iterative nature and the additional prior information used (finite support, positivity, pure-phase object), the algorithm is able to recover missing frequencies. In all but the mHIO reconstructions, artifacts can be recognized in the power spectra as oscillations, corresponding to the zero crossings of the CTF. Only the mHIO reconstruction shows sufficient correction of these oscillations. Experimental parameters necessary to record the holograms of the cells are shown in Table 1 ▸. At around 0.3 periods per pixel, a crossover to the noise plateau is observed in the power spectrum, corresponding to a half-period resolution of less than 50 nm.

Fig. 4 ▸ shows the performance of multi-distance phase-retrieval algorithms. Four holograms are recorded at slightly different Fresnel numbers, one of which is shown in Fig. 4 ▸(*a*). The results obtained with TIE and holographic reconstructions are not shown, as they only require a single image. When comparing the four multi-distance approaches, only slight differences can be observed. In particular, the CTF approach (Fig. 4 ▸ *c*) still shows some image artifacts which appear as fringes around the cell. The two iterative methods Figs. 4 ▸(*d*) and 4 ▸(*f*) show slightly different absolute values. For the holo-TIE approach, the two centre distances having the smallest difference in Fresnel number have been used in combination with the low-frequency phases in the detector plane, as calculated from the CTF approach. To this end, a pure-phase wave 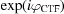 was built, propagated to the detector plane using the Fresnel propagator 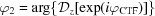 and finally used as a low-frequency detector phase map as in equation (8). By this approach, the residual twin image artifacts can be removed, without the need for a computationally expensive iterative reconstruction.

### 3.2. Three-dimensional phase retrieval   

**Figure 5:**
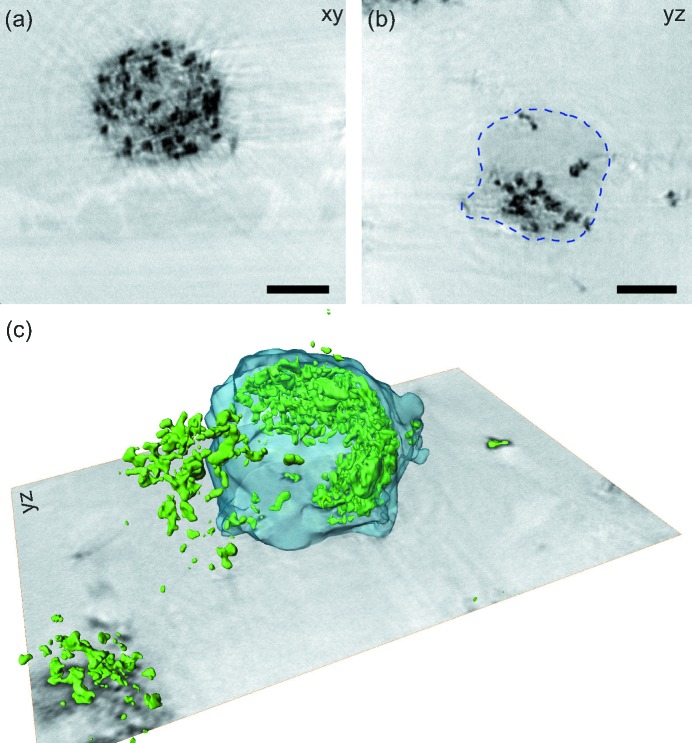
Tomography result of an isolated macrophage which was labelled with barium sulfate and osmium tetroxide. (*a*) Orthoslice through the plane perpendicular to the tomographic axis. Besides the cell with its internal contrast particles, the Petri dish and the resin used for embedding can be resolved too. (*b*) Orthoslice coplanar to the projection direction. (*c*) Three-dimensional rendering of the data set, showing barium particles in green and the homogeneous osmium inside the cell in half-transparent blue. Phase retrieval for all projections was carried out using the CTF approach with four distances, followed by a tomographic alignment procedure. Scale bars denote 5 µm.

To obtain three-dimensional reconstructions, the dried cell preparations shown above were not suitable as the cell flattens during the drying process. Hence, a resin-embedding procedure was used. Additionally, the cells were stained with OsO_4_ to increase the contrast of lipids and thereby the visibility of the cell body. Fig. 5 ▸ shows the results after reconstructing every single projection with the CTF approach using four distances, followed by filtered back-projection (FBP). To obtain a high-quality reconstruction, residual motion in the data set was removed by enforcing tomographic consistency (Guizar-Sicairos *et al.*, 2015 ▸; Töpperwien *et al.*, 2016 ▸). The reconstructed slice in Fig. 5 ▸(*a*) shows the Petri dish with lower density on the bottom (brighter grey), the slightly denser and thus darker resin, the medium dark (due to the OsO_4_) cell body and the BaSO_4_ particles (black). Next to the two orthoslices shown in Figs. 5 ▸(*a*) and 5 ▸(*b*), a three-dimensional rendering is shown in Fig. 5 ▸(*c*), in which individual barium particles are segmented based on a global threshold. The outline of a single cell is segmented using the magic wand tool of *Avizo*, followed by morphological operations. The overall three-dimensional structure is in good agreement with the cell shape of macrophages observed in lung tissue of mice (Krenkel *et al.*, 2015 ▸) and in two-dimensional visible light microscopies. However, the OsO_4_ stain is far from *in situ* conditions so we turned to a different preparation and embedding of cells, as presented in the next section.

### 3.3. Imaging of hydrated cells   

**Figure 6:**
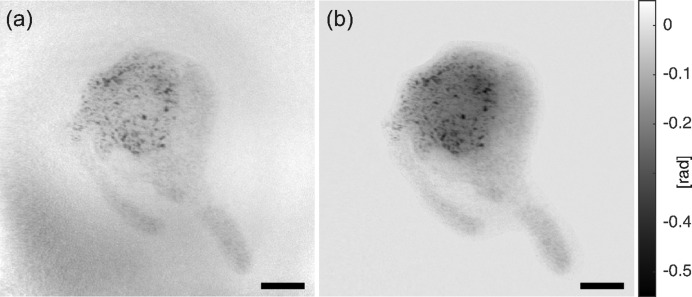
Phase distributions of macrophages in PBS, labelled with gold-enhanced antibodies (anti-CD68). The holograms were recorded under non-optimal illumination conditions, compromising the quality of the phase retrieval. (*a*) Phase distribution reconstructed using the CTF-based multi-distance approach, which amplifies the artifacts present in the hologram. (*b*) Iterative multi-distance HIO reconstruction, where a manually determined support area is enforced, sufficient to suppress the artifacts. From Krenkel (2015 ▸). Scale bars denote 5 µm.

For a close-to-native sample state, the cells were incubated in a liquid chamber (ibidi GmbH, Germany) and a gold nanoparticle stain was used to increase the weak contrast of the cells. However, as for this particular beamtime a tapered waveguide was chosen, the illuminating empty beam still exhibits slight artifacts, which may be amplified by the phase-retrieval step. Fig. 6 ▸ shows two reconstructions using the CTF approach in Fig. 6 ▸(*a*) and the mHIO approach with manually determined support area in Fig. 6 ▸(*b*). The detailed structure of the macrophage can be observed in both cases; however, the CTF phase map shows low-frequency variations that may limit quantitative analysis like tomography. These artifacts are already present in the hologram, but less pronounced and are transferred to the low frequencies by the phase-retrieval step. Contrarily, the iterative reconstruction is able to compensate for these variations, in particular due to the use of soft projections, which acts as an implicit regularization to the reconstruction. However, this comes at the price of additionally required prior knowledge and increased computation time. Note that not only is the uniformity of the background ensured but also the low-frequency structure inside the cell appears much more plausible in the iterative reconstruction, which is important for further analysis.

**Figure 7:**
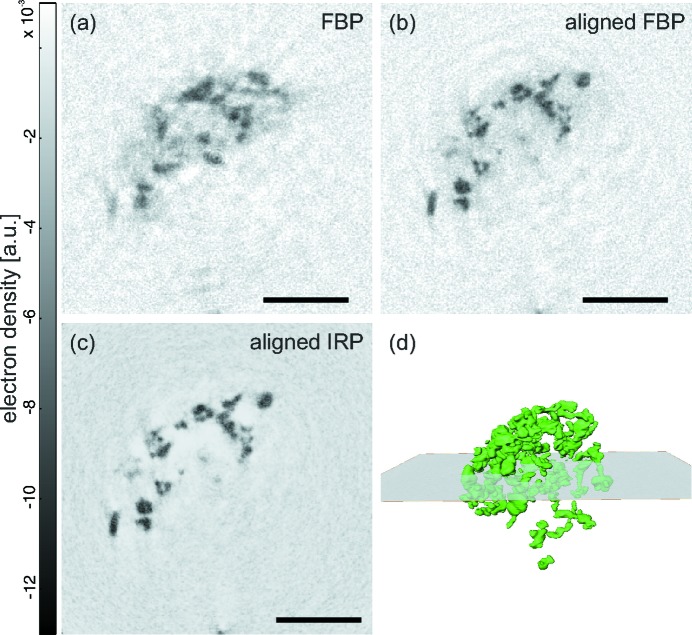
(*a*) Orthoslice through a macrophage, reconstructed by filtered back-projection from projections which were phased by single-distance CTF, without any tomographic alignment processing. (*b*) The same slice as in (*a*) but with tomographic consistency enforced by alignment algorithms. (*c*) Combined iterative reprojection phase retrieval (IRP) of the cell, as described in the main text. (*d*) Three-dimensional rendering of the IRP reconstruction. From Krenkel (2015 ▸). Scale bars denote 5 µm.

Motivated by the excellent performance of iterative algorithms even for difficult data sets, which suffer from strong noise and inconsistence, we then addressed three-dimensional reconstruction of a single hydrated cell inside a capillary. To this end, we have in mind that iterative algorithms should also enhance the quality of three-dimensional reconstructions. A BaSO_4_-labelled macrophage was embedded in agarose and transferred to the capillary while the agarose was still fluid. Upon gelation, the agarose then fixes the cell in space. However, this kind of preparation led to the problem that, with the X-ray beam turned on, the sample started to move along the direction of the capillary, presumably due to beam-induced heating and melting of the agarose. In a three-dimensional reconstruction this results in a blurring of the barium particles inside the cell, as can be clearly seen in Fig. 7 ▸(*a*), which shows the result after tomographic reconstruction, with each projection subjected to CTF phase retrieval. By enforcing tomographic consistency (Guizar-Sicairos *et al.*, 2015 ▸; Töpperwien *et al.*, 2016 ▸), these motions can be compensated for, yielding a much sharper reconstruction (Fig. 7 ▸ *b*). However, some typical fringe artifacts can still be recognized, which are caused by the fact that only a single distance was used for the CTF reconstruction. Using more than a single distance was impeded by the fact that also the sample orientation changes over a larger time frame. This could be neglected in a single-distance tomographic scan, which took only a few minutes. Applying the mHIO reconstruction, on the other hand, was not easily possible, as dirt and other cells outside the field of view traversed the projections in some angles. Therefore, we turned to the combined iterative reprojection phase-retrieval (IRP) algorithm, in which a low-frequency overlap of neighbouring projections is used to stabilize phase retrieval (Ruhlandt *et al.*, 2014 ▸; Ruhlandt & Salditt, 2016 ▸). Fig. 7 ▸(*c*) shows the resulting reconstruction. It can be clearly seen that the signal-to-noise ratio is improved and that twin image artifacts were suppressed. However, the very weak contrast of the underlying unstained cell body is not sufficient to observe the native cell body. In particular, if the cell moves differently from the barium particles, it will be further blurred as tomographic alignment is based on the strong contrast of the BaSO_4_ particles.

## 4. Discussion and conclusion   

In this work we have benchmarked propagation-based phase-contrast imaging of biological cells, in particular mouse alveolar macrophages, in two and three dimensions, by comparison and optimization of different phase-retrieval approaches. In particular, we have demonstrated an improved holo-TIE algorithm, based on a four-distance recording, and a weighted phase map for low and high spatial frequencies. Importantly, the range of applicability of this approach is extremely high, as neither restrictive assumptions on the object’s optical properties (such as homogeneity or weakly varying phase) nor restrictive prior information such as support or sparsity is required. Further, the algorithm can be applied over the full range of Fresnel numbers and not only in the direct-contrast regime. Fig. 4 ▸ nicely demonstrates that the image quality of the holo-TIE approach surpasses conventional CTF and almost reaches that of iterative algorithms (mHIO, IRGN), which are computationally more complex and also require prior information.

At the same time, our results show that iterative algorithms optimized for phase retrieval of single- or multi-distance data sets are the method of choice if the data suffer from low signal-to-noise ratio or from inconsistencies. The latter can easily result from drifts of the illumination system or motion of hydrated cells. Prior information, in particular on the support of the cell, in combination with soft projections are best suited to address such data sets. Importantly, the iterative approach can be carried out in an all-at-once approach combining phase retrieval and tomographic reconstruction. Fig. 7 ▸ shows the superior results for a tomographic recording of a hydrated cell which suffered from object motion during the scan.

Regarding the reconstructed cellular shape of macrophages and the interior aggregates of the barium sulfate contrast agent, the results corroborated those observed by tomography of macrophages in lung tissue (Krenkel *et al.*, 2015 ▸). Reciprocally, the image quality demonstrated here for a number of preparations and algorithms should all be more or less also achievable in thick tissues, which would exploit the unique advantages of hard X-ray imaging.

In summary, we can conclude that phase retrieval is no longer the bottleneck for propagation-based phase-contrast imaging, and that both recent iterative algorithms as well as refined TIE solutions have led to enabling progress. The persisting challenges are now more related to suitable sample preparation and environment, as well as to the stability (optical and mechanical) of the recording. To this end, we have compared many different settings and preparations in this work.

The desired unlabelled and hydrated state of biological cells, which is a unique potential of hard X-ray imaging, is most challenging. At the given experimental setting of low-dose recordings, the native electron density at the organelle level is poorly transferred. From recent ptychographic results (Giewekemeyer *et al.*, 2015 ▸) and from CDI of biological cells, we know that this is different for high-dose recordings, but this requires cryogenic fixation (frozen hydrated samples) to prevent structural disintegration by radiation damage. Alternatively, phase-contrast imaging in cone-beam geometry at high magnification is ideally suited for single-shot ultrafast imaging. In contrast to the data shown here, a projection image of the instantaneous electron density which would be unaffected by sample motion and drift could be recorded.

## Figures and Tables

**Table 1:** Overview of experimental parameters used for two-dimensional imaging Exposure times are stated per image. The waveguides were a crossed multilayer waveguide with 59 nm guiding core (59 nm) and a bonded air/silicon waveguide in tapered geometry (taper). For some images the effective pixel size is resampled by a factor of 2 to increase the signal-to-noise ratio.

	Fig. 3 ▸	Fig. 4 ▸	Fig. 5 ▸	Fig. 6 ▸	Fig. 7 ▸
Sample	Dried	Dried	Epon	ibidi	Capillary
Effective pixel size (nm)	29.3	22.9	2 × 24.6	2 × 25.0	2 × 25.4
Min. 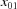 (mm)	23.0	18.0	19.3	19.6	19.7
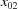 (m)	5.13	5.13	5.12	5.12	5.07
Energy (keV)	13.6	13.6	8.0	8.0	13.8
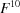	0.042	0.033	0.020	0.021	0.037
Waveguide	59 nm	59 nm	Taper	Taper	59 nm
Number of distances	1	4	4	5	1
Number of projections	—	—	2040	—	360
Angular range (°)	—	—	360	—	360
Exposure time (s)	10	10	0.2	2	1
